# Unusual endoscopic remission in an anti‐tumor necrosis factor refractory case of severe ulcerative colitis during upadacitinib therapy

**DOI:** 10.1002/jpr3.70125

**Published:** 2025-12-09

**Authors:** Carine A. Halaby, Charlotte F. Kim, Richard Kellermayer

**Affiliations:** ^1^ Division of Pediatric Gastroenterology, Texas Children's Hospital Baylor College of Medicine Houston Texas USA; ^2^ Department of Pathology, Texas Children's Hospital Baylor College of Medicine Houston Texas USA

**Keywords:** inflammatory bowel disease, JAK inhibitors, postinflammatory submucosal fibrosis

A 14‐year‐old female with ulcerative pancolitis (UC pancolitis), diagnosed by endoscopy at an outside hospital, was started on infliximab (IFX) 300 mg (5 mg/kg). After initial improvement, she relapsed severely (Pediatric Ulcerative Colitis Activity Index [PUCAI]^1^ = 70) 1 week postfirst dose. She was admitted for accelerated IFX (5 mg/kg at 1 week, then 10 mg/kg at 4 weeks) plus IV steroids but remained moderately to severely active after the third IFX (level 9.5 µg/mL; no antibodies). Colonoscopy 8 days after the third IFX (5 weeks from initiation), while on steroids, showed severe pancolitis (Mayo^2^ 2–3) with extensive pseudopolyposis (Figure 1A); magnetic resonance (MR) enterography confirmed diffuse pancolitis. Upadacitinib (UPA 30 mg daily) was started as third‐line therapy,^3^ yielding rapid improvement. Vedolizumab (VEDO 300 mg every 8 weeks) was added 1 week later as a bridge target, with UPA continued. Steroids were tapered from 40 mg over 25 days from UPA initiation. After 2.5 months of dual therapy, reducing UPA to 15 mg caused relapse; 30 mg daily was resumed. She has since maintained >2 years of remission on dual (UPA/VEDO) therapy (patient preference), with normalized fecal calprotectin (43 µg/g). Surveillance colonoscopy at 1.5 years postdiagnosis (1 year in remission) showed fibrotic ridges, normal mucosa, and no active inflammation (Figure 2A); histology showed subepithelial collagen deposition (Figure 2B).

**Figure 1:**
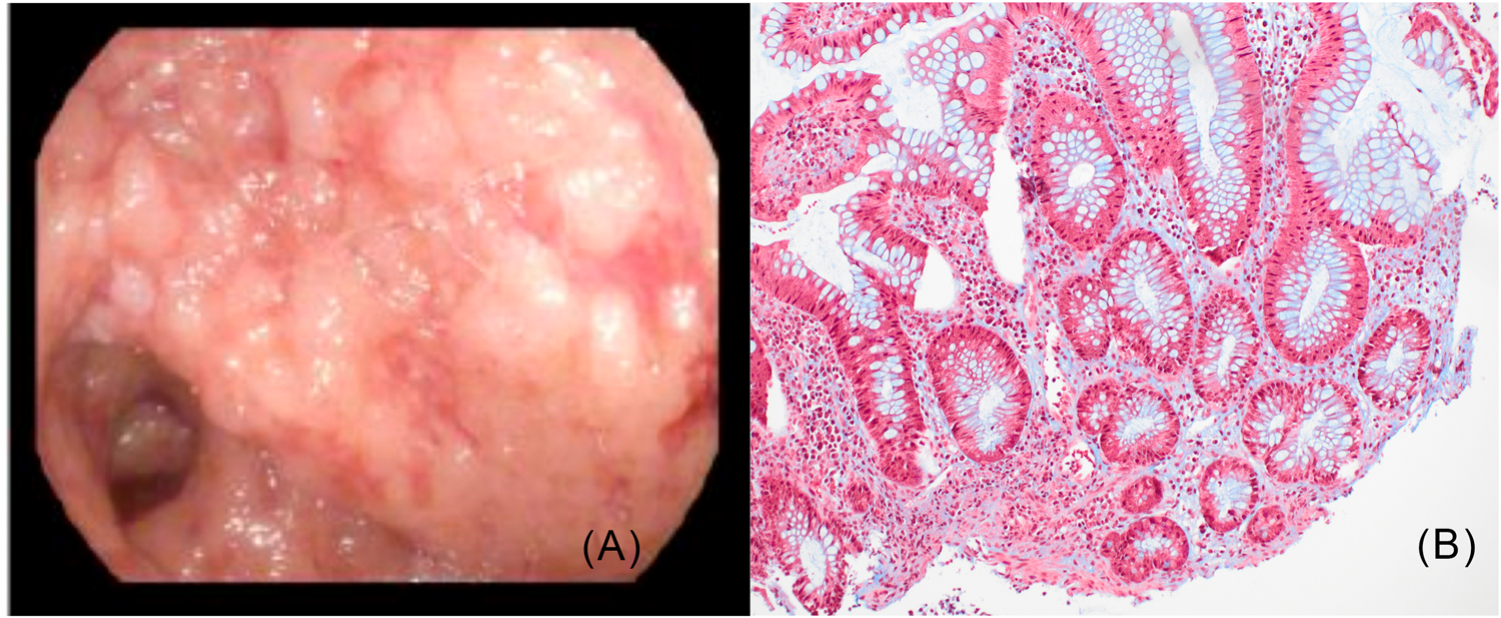
(A) Initial colonoscopy at our institution demonstrating severe inflammation consistent with Mayo 2–3 colitis, characterized by friability, ulceration, and pseudopolyps. (B) Corresponding histology with Masson's trichrome stain showing lamina propria edema without collagen deposition.

**Figure 2:**
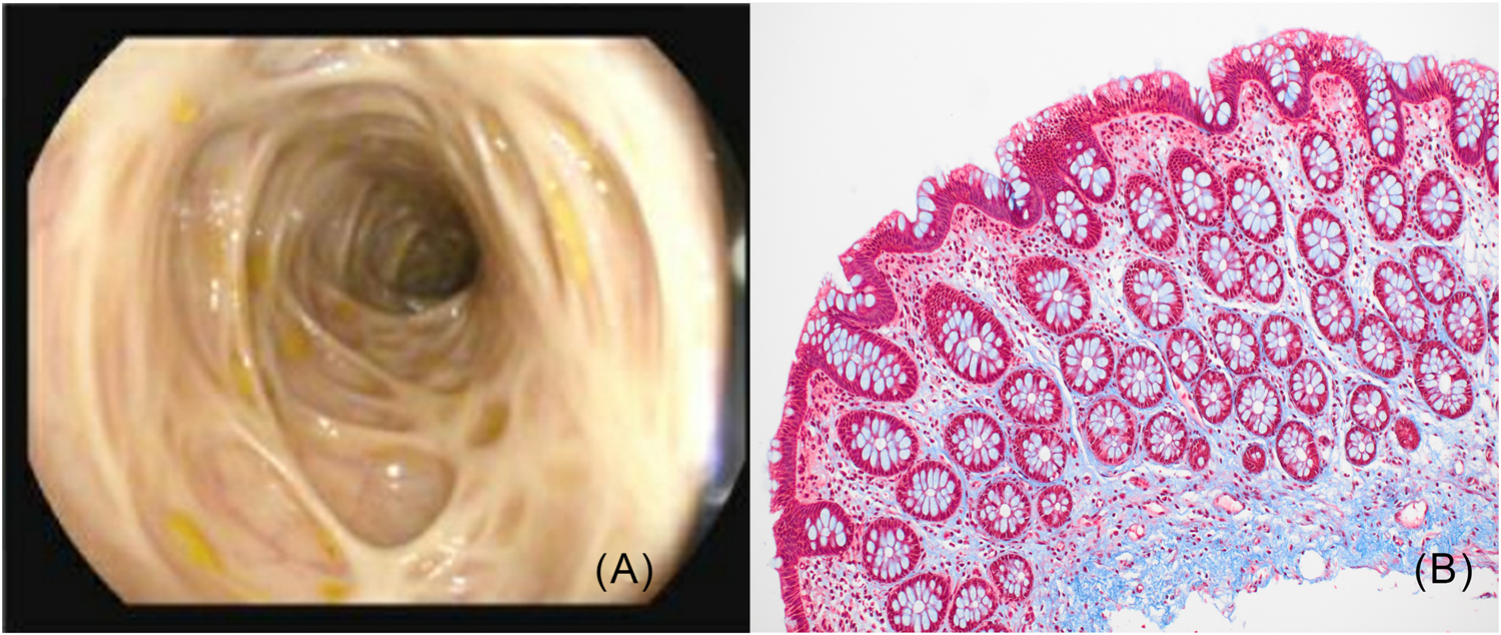
(A) Surveillance colonoscopy with coalescing fibrotic ridges throughout the colon, with endoscopically normal‐appearing mucosa. (B) Histologic section stained with Masson's trichrome demonstrating basally‐oriented collagen deposition within the lamina propria consistent with fibrosis, notably without any features of chronic colitis (i.e., no crypt distortion, crypt branching, crypt dropout, or basal lymphoplasmacytosis).

UPA is an emerging off‐label option for acute severe, anti‐tumor necrosis factor (TNF)‐refractory pediatric UC, with growing evidence of efficacy.^4^ Historically, such cases commonly progressed to colectomy.^5^ We propose that fulminant, treatment‐ refractory UC may represent a distinct inflammatory bowel disease (IBD) subtype,^6^ presenting clinically as UC pancolitis but exhibiting more extensive transmural involvement and submucosal fibrotic characteristics, as observed by Gordon et al.^7^ Notably, the presence of fibrosis and transmural changes‐ features classically associated with Crohn's disease (CD)‐raises a diagnostic dilemma. However, recent studies^8^ show that these features can occur in UC, especially in pediatric and long‐standing cases, and do not necessarily justify reclassification as CD in the absence of other distinguishing features such as granulomas, skip lesions, or small bowel involvement. This highlights potential limitations of current IBD classification and possible need for further refinement. Our case supports this predicament and underscores the importance of closely monitoring patients with severe, anti‐TNF refractory UC who respond to UPA “rescue” or “third‐line” therapy.

## CONFLICT OF INTEREST STATEMENT

The authors declare no conflicts of interest.

## ETHICS STATEMENT

An informed verbal consent was obtained from patient and parent before submission of the deidentified image case report.
